# Ethically Driven and Methodologically Tailored: Setting the Agenda for Systematic Reviews in Domestic Violence and Abuse

**DOI:** 10.1007/s10896-023-00541-7

**Published:** 2023-04-03

**Authors:** Karen Schucan Bird, Nicola Stokes, Martha Tomlinson, Carol Rivas

**Affiliations:** 1 Social Research Institute, University College London, 10 Woburn Sq, London, WC1H 0NR UK; 2 SafeLives, Suite 2a, Whitefriars, Lewins Mead, Bristol, BS1 2NT UK

**Keywords:** Systematic review, Ethics, Methodology, Informal social support, Research integrity

## Abstract

**Purpose:**

Systematic reviews have an important, and growing, role to play in the global evidence eco-system of domestic violence and abuse. Alongside substantive contributions to knowledge, such reviews stimulate debates about ethical reviewing practices and the importance of tailoring methods to the nuances of the field. This paper aims to pinpoint a set of ethical and methodological priorities to guide and enhance review practices specifically in the field of domestic abuse.

**Method:**

The five Pillars of the *Research Integrity Framework* (ethical guidelines for domestic abuse research) are used to interrogate the systematic review process. To do so, the *Framework* is retrospectively applied to a recently completed systematic review in domestic abuse. The review included a rapid systematic map and in-depth analysis of interventions aimed at creating or enhancing informal support and social networks for victim-survivors of abuse.

**Results:**

Ethical and methodological priorities for systematic reviews in domestic abuse include (1) Safety and wellbeing: maintaining the wellbeing of researchers and stakeholders, and appraising the ethics of included studies, (2) Transparency/ accountability: transparent reporting of research funding, aims and methods together with explicit consideration of authorship of outputs, (3) Equality, human rights and social justice: developing diverse review teams/ Advisory groups, and review methods that aim to search for, and report, diverse perspectives. Considering researcher positionality/ reflexivity in the review, (4) Engagement: collaboration with non-academic stakeholders and individuals with lived experience throughout the review process, (5) Research Ethics: independent ethical scrutiny of systematic review proposals with input from researchers with expertise in systematic reviews and domestic abuse.

**Conclusion:**

Additional research is required to comprehensively examine the ethics of each stage of the review process. In the meantime, attention should be given to the underpinning ethical framework for our systematic review practices and the wider research infrastructure that governs reviews.

## Introduction

Systematic reviews have an important, and growing, role in the global evidence eco-system of domestic violence and abuse (DVA). Systematic reviews are reviews of ‘existing research using explicit, accountable rigorous research methods’ (Gough et al., 2017b, p. 2). Over the past fifteen years, there has been a significant expansion in the conduct and publication of systematic reviews in the field of DVA (MacGregor et al., 2014), spanning a wide range of topics from perpetrator programmes (for example, see Bell & Coates, 2022) to children’s experiences of domestic abuse (Noble-Carr et al., 2021). This body of review level evidence has a critical role to play in developing effective policies and programmes to prevent, reduce and mitigate the effects of DVA across multiple sectors, including public health, criminal justice, and social work (Addis & Snowdon, 2021).

Alongside substantive contributions to knowledge, systematic reviews in DVA stimulate wider debates about ethical reviewing practices and the importance of tailoring methods to the nuances of the field. Such reviews have evolved over time to respond to the shifting demands of policy/ practice and adapt to a growing, but challenging, evidence base. Early reviews of interventions in DVA followed standard methodologies for ‘what works/ effectiveness’ reviews (using comparative, quantitative studies to test impacts of interventions) (see Gough & Thomas, 2017; Munn et al., 2018) to reveal a severe lack of ‘evidence of suitable quality’ (such as Wathen & MacMillan, 2003). More recently, intervention reviews have begun to develop innovative methodologies to maximise the contributions of the evidence base, despite the limitations, and draw inferences for applied contexts (such as Rivas et al., 2019; Trabbold et al., 2020). Yet, reviews of DVA interventions continue to face a number of challenges as the research landscape includes few high-quality, well-designed trials, an absence of standardised measures, and limited relevance for DVA policy/practice questions (Bell & Coates, 2022; Feder et al., 2011; Tarzia et al., 2017 ). This literature includes small sample sizes, short term follow-up, an over reliance on official sources of data (such as police records) and narrow conceptions of DVA (primarily focusing on physical violence) (Bell & Coates, 2022). Moreover, the studies tend to report data on relatively homogeneous population groups (predominantly heterosexual, female, white, adult samples) (Addis and Snowman, 2021) from specific geographical areas (North American studies dominate the field) (Gregory et al., 2017; Trabold et al., 2020). Nevertheless, DVA research also offers possibilities for systematic reviewers. The field is growing, underpinned by a rich scholarship on ethical considerations (Bender, 2017) and includes a high number of methodological robust qualitative studies, although they tend to be poorly reported (Arai et al., 2021; Meyer et al., 2020; Noble-Carr et al., 2021). A further strength rests with the collaborative and transdisciplinary nature of DVA research, with a significant proportion of studies undertaken and published through non-academic channels, and typically dispersed across disciplines and sectors (Bender, 2017; Konya et al., 2020). Such characteristics present numerous challenges and opportunities for researchers who seek to undertake relevant and useful systematic reviews for improving policy and practice in DVA (Tarzia et al., 2017). Yet, there is limited methodological debate about whether and how to tailor review methods to the field of DVA. Further, the centrality of ethical issues has remained marginal to methodological developments in reviews (Suri, 2020). This article take steps to address these gaps by aiming to pinpoint a set of ethical and methodological priorities to guide and enhance review practices specifically in the field of DVA. This endeavour aligns with the broader imperative to develop standards of conduct to promote and maintain quality standards in reviews (Higgins et al., 2022; White et al., 2018a).

## Methods

This articles uses the *Research Integrity Framework* (*RIF*) (Women’s Aid, 2020), supplemented by additional themes identified in wider literature (such as Bender, 2017; Ellsberg & Heise, 2002; World Health Organization, 2001) to identify ethical priorities in DVA research. The *RIF* aims to sketch out ‘what good research practice relating to DVA looks like’ (Women’s Aid, 2020: 2) and was collaboratively developed by DVA practitioners from non-governmental organisations together with academics in the UK. The *RIF* uses five pillars to ‘highlight the key aspects of research in this field’ (Women’s Aid, 2020: 5): (1) Safety and wellbeing, (2) Transparency/ accountability, (3) Equality, human rights, and social justice, (4) Engagement, (5) Research ethics. Drawing on feminist research practice, the *RIF* offers a useful starting point for analysing DVA specific methodologies and presents an initial attempt to develop standards of (primary) research conduct in this field. In doing so, the *RIF* offers a series of checklists to support best practice and aims to assist policy makers/ commissioners in appraising DVA research. Indeed, there are examples of the *RIF* being used in policy orientated research teams (such as the Domestic Abuse Commissioner for England and Wales).

In this article, the *RIF* is applied to a recently completed systematic review in the field of domestic violence and abuse (Schucan Bird et al., 2022) . This review, funded by the Economic & Social Research Council (ESRC), as part of UK Research & Innovation’s rapid response to Covid-19, was a collaboration between university academics and researchers from a domestic abuse organisation. An Advisory group composed of individuals with lived experience, frontline DVA service providers, and DVA specialists was created to feed into the project at three key points. The systematic review examined empirical research on informal social support interventions (‘activities designed to change the existing quality, level, or function of an individual’s personal social network or to create new networks and relationships’, Budde & Schene, 2004, p. 342). The review followed a two-stage approach, beginning with a systematic map of qualitative and quantitative studies (complying with key characteristics associated with ‘scoping study’, ‘systematic map’, and ‘evidence map’ in Snilstveit et al., 2016). The second stage included a mixed methods review (Grant & Booth, 2009) that was informed by an EPPI Centre approach (Gough et al., 2017a; Hong et al., 2020).

With a specific focus on interventions in DVA, the case review follows on from traditional systematic reviews that assess the evidence base for interventions (Munn et al., 2018). As such, the methods of the first stage of the review were guided by standards of conduct tailored towards reviews of intervention studies (White et al., 2018b, 2020) and included an element of pre-specification that was expected of such review types (such as searching, and inclusion criteria established *a priori*). Similarly, the review questions, in the second stage, asked about the effectiveness of DVA interventions and so included elements common to wider ‘What works’ reviews (Gough & Thomas, 2017). However, the focus and methods of the case review also extended beyond traditional ‘effectiveness reviews’ by asking wider questions about the interventions and including diverse study designs (Schucan Bird et al., 2022) . This reflected the EPPI-Centre commitment to adjusting review methods to respond to questions in meaningful and useful ways, drawing on a selection of ‘different tools within our toolbox of review approaches’ (Gough & Thomas, 2017, p. 52). Therefore, the case review did not faithfully embody one type of review (Munn et al., 2018) but included a combination of different review types (systematic map and mixed method syntheses). This reflects the variety of reviews undertaken in the field of domestic abuse including qualitative evidence syntheses (such as Sinko et al., 2021) and ‘scoping reviews’ of research (such as Aljomaie et al., 2022).

As ‘reviews can vary on many dimensions and that these may go beyond branded types of review’ (Gough & Thomas, 2017, p. 49), other characteristics of the case example shared similarities with the wider body of DVA reviews: a broad conceptualisation of the problem and rapid imperative. The case review focused on interventions targeting DVA, broadly conceptualised (similar to recent DVA reviews such as Anderson et al., 2021; Trabold et al., 2020) and used techniques to expediate the review process. This aligns with the imperative to undertake rapid research in the field of DVA, especially during the pandemic, to develop ‘actionable’ findings that can be translated into policy and practice recommendations (Richardson et al., 2020). The review aimed to produce research outputs that were ‘useful, useable and used’ (Graham et al., 2019, p. 1) and so the methods of the review were shaped by an ethical imperative to improve outcomes for victim-survivors of DVA. The execution of common review types, similarities with DVA reviews of interventions combined with a heightened awareness of ethical issues makes this review a suitable example. The *RIF* was applied retrospectively to this case review and each of the Pillars are considered below.

## Findings and Discussion

### Pillar 1: Safety and Wellbeing

The *RIF* defines safety and wellbeing in terms of the maintenance of the ‘safety, both physical and emotional, of research participants, and researchers themselves, within the research process’ (Women’s Aid, 2020: 5). The protection of research participants has long been a concern for scholars of DVA (Burgess-Proctor, 2015; Clark & Walker, 2011; Mulla & Hlavka, 2011) but the systematic review process does not include direct research participants. Therefore, the remit of safety and wellbeing in the case review relates to the participants in the primary studies included in the review, the researchers themselves and the stakeholders involved in the Advisory Group. These will now be considered.

#### Participant Safety in Primary Studies

Common to systematic reviews, the case review did not examine whether the included studies assured the safety and wellbeing of research participants (Elia et al., 2016). The review used a quality assessment tool to appraise the methods of included studies but this did not include ethical criteria (Hong et al., 2018). Neither conduct nor reporting guidelines for systematic reviews, such as Cochrane Collaboration (Higgins et al., 2022) or PRISMA (Page et al., 2021a) stipulate that reviews should assess or report the ethical approval of the included primary studies. Yet, there are growing calls to do so as part of wider efforts to address research misconduct (Elia et al., 2016; Vergnes et al., 2010; Weingarten et al., 2004). Assessing the ethics of included studies is highly appropriate for systematic reviews in DVA, given the centrality of the safety of participants for research in this field (Women’s Aid, 2020).

#### Researcher Wellbeing

Typical of academic projects in gender-based violence, the study did not explicitly identify the support needs of the researchers (Nikischer, 2019; Schulz et al., 2022). Yet, maintaining the safety and wellbeing of researchers is ‘paramount and should guide all project decisions’ in the field of DVA (World Health Organization, 2001, p. 10). Systematic reviewers do not collect data from victim-survivors so potentially have a different level of exposure to distressing or trauma content compared with primary researchers. Nevertheless, secondary data collection and analysis including sensitive topics can still involve a degree of ‘emotional work’ with associated risks to researcher wellbeing (Hanna, 2019; Jackson et al., 2013). Several strategies may mitigate potential harms, as illustrated by the case review. Reviewers had access to support services provided by the institutions to which they were affiliated. The domestic abuse organisation, SafeLives, provided tailored and responsive support services for researchers (see, for example, the Clinical Supervision Guidelines SafeLives, 2022). The academic institution provided access to a limited number of sessions from an external, generic mental health provider. The wider literature highlights that such services are insufficient and unable to respond to the impacts associated with working in the field of DVA (Schulz et al., 2022). New researchers may feel particularly reluctant to seek external support for fear of compromising their professional identity (Hanna, 2019). Other strategies organically emerged in the case review including informal peer-to-peer support and the emergence of collective caring (Neale, 2013). Support from colleagues in DVA, ‘turning to others who get it’, is considered to be an important pillar for researcher wellbeing alongside, or in the absence of, tailored institutional mechanisms (Schulz et al., 2022) but this may not always be available to researchers (Jackson et al., 2013). Aside from informal coping strategies, studies recommend that institutional and system-wide responses are needed to provide ethical infrastructure to maintain the wellbeing of DVA researchers. This includes training in researcher self-care and extending university ethics protocols to protect researcher well-being in potentially traumatic fields (Cullen et al., 2021; Nikischer, 2019). Whilst systematic review guidelines or ethical discussions currently overlook issues associated with reviewer welfare (Higgins et al., 2022; such as Suri, 2020; Vergnes et al., 2010), using established strategies from primary research can ensure that secondary researchers are better protected when engaging with difficult and potentially distressing data (Hanna, 2019).

#### Wellbeing of Advisory Group

Much discussion of ethics in studies of DVA centres on maintaining the physical and emotional safety of victim-survivors, primarily as research participants (Bender, 2017; Ellsberg & Heise, 2002; Women’s Aid, 2020). These discussions also have applicability to stakeholder involvement in systematic reviews. The case review provided the Advisory group, including individuals with lived experience, with Terms of Reference and a Privacy Notice to garner informed consent and share the protocols regarding anonymity/ confidentiality. Whilst these documents established ground rules for involvement, the case example identified two further ethical issues. First, the team did not explicitly consider the potential for inadvertently causing harm or distress to stakeholders (World Health Organization, 2001). In the case review, not all members of the research team had prior experience of working in the field of DVA and/ or sufficient understanding of the sensitivities in this area. This may potentially have increased the risk of harm to Advisory group members during the review process. Discussion in Advisory group meetings in the case review, for example, prompted individuals with lived experience to recall difficult experiences and some questions may have been phrased insensitively. The make-up of systematic review teams, typically large and constituted by methodologists and topic experts (Oliver et al., 2017), means that not all reviewers will have substantive knowledge of DVA. To minimise the potential for harm, DVA reviews should ensure that all researchers have sufficient experience or training in the field, including a basic introduction to DVA and an opportunity to explore their own biases and fears (World Health Organization, 2001).

Second, ethical considerations pertaining to the wellbeing of the Advisory group may be shaped by the type of systematic review and the extent to which methods/ concepts/ data are pre-specified. Stakeholders in reviews with highly specified methods and concepts (such as ‘What works’ reviews, see Gough and Thomas, 2017) should have a reasonably complete understanding of the scope of the review and the likely data they will encounter. Such information can therefore be used to inform stakeholders’ decisions to provide consent for involvement. Reviews with more inductive approaches, however, mean that Advisory groups may not have sufficient information at the outset of the review to be able to make an informed judgement. The concepts and data used in the synthesis of the case review, for example, emerged through the review process yet informed consent was only sought at the start of the project. Therefore, it is helpful to recognise consent as an ongoing process and establish ethical protocols to ensure stakeholders can withdraw their participation at any point in the research process (Neale, 2013).

Whilst the wellbeing of individuals with lived experience was partially addressed in the case review, there was a lack of explicit concern for other stakeholders (such as service providers or policy colleagues). Pillar 1 of the *RIF* only focuses on the safety and wellbeing of a specific group of research participants: those directly involved in the data collection. Extending our understanding of participants to include all individuals involved in the research, ‘however tangential the involvement may be’, can recognise the importance of the wellbeing of all stakeholder groups (Neale, 2013, p. 7). This ‘stakeholder ethics’ aligns with wider strategies in the DVA sector that recognise that anyone working in the field may have direct experience of abuse/ trauma and so need recourse to support services (SafeLives, 2018).

### Pillar 2: Transparency/ Accountability

Transparency/ accountability in the *RIF* refers to the importance of being explicit about the entire research process, including ‘who is doing the research?’, to enable policy makers to assess the merit of the research. This section will focus on issues relating to the reporting of the funder/ commissioner of the research, the research aims and methods, and review outputs (Women’s Aid, 2020: 8).

#### Research Funding

Pillar 2 of the *RIF* highlights the importance of reporting research funding ‘so that the reader is clear about any vested interests’ (Women’s Aid, 2020: 9). The case example was contractually obliged, by the public funder (UKRI), to explicitly state the source of funding in any publications or publicly facing outputs. Once the funding had been secured, the project was relatively autonomous and without explicit vested interests. The ethics of research funding, however, extends beyond concerns about vested interests to include the role and purpose of public resources for systematic reviews in DVA.

In most countries, funding for research is mainly channelled towards primary research with comparatively few resources provided for systematic reviews or strategies to maximise research use. This balance of resources raises ethical concerns as such funding structures arguably fail to maximise existing research knowledge and/ or use this research to inform policy and practice decisions (Gough et al., 2019). Against this backdrop, we have seen renewed political commitments to use evidence over the past two decades with research synthesis playing a central role (Breckon & Gough, 2019). The publicly funded network of ‘What Works Centres’ in the UK, for example, have designated systematic reviews as ‘the best available research evidence’ and DVA systematic reviews have been commissioned and catalogued as part of this process (e.g., The Crime Reduction Toolkit). ‘What Works’ reviews in DVA have highlighted the rich and abundant field of primary research and the efficient and valuable contribution that syntheses offer for policy making (such as Addis & Snowdon, 2021). Yet, there are also concerns that a narrow focus on ‘What works’ overlooks the wider array of review questions that may be important for stakeholders (Munn et al., 2018). Moreover, a focus on ‘effectiveness’ and ‘impact’ may control or limit research agendas through ‘themed’ or ‘directed’ funding streams that prioritise particular forms of ‘useful’ research over others (Chubb & Reed, 2018). This agenda may present a challenge to ‘scholarly moral conduct’ by compromising integrity and shaping studies to fit into current policy agendas (Chubb & Watermeyer, 2017). These debates have resonance for the research agenda in DVA, where the extent and nature of funding fluctuates with policy priorities (See Auchter & Backes, 2013 for an example from the USA) and there has always been a ‘moral obligation on researchers’ to ensure that DVA findings are useful and used for advocacy, policy, and intervention (Ellsberg & Heise, 2002, p. 1602; Women’s Aid, 2020). In the UK, public funding streams have provided welcome opportunities to develop appropriate and effective studies into violence against women, and embed research in the political agenda (Harwin, 2006). However, there are concerns that such an agenda may marginalize and overlook particular communities (such as disabled women, LGBTQ + communities, men. See Bates & Douglas, 2020; Harwin, 2006) as funding may not be offered to research with more specialised reach and low impact potential (Chubb & Reed, 2018).

The review provides an interesting case example because it was funded *despite* sitting outside the traditional remit of government agencies or policy priorities in the UK. The focus on informal support for victim-survivors has been hitherto overlooked by policy (e.g., Domestic Abuse Act, 2021, does not explicitly refer to informal supporters) and practice (Goodman & Smyth, 2011). Indeed, funding for the case example, a responsive stream related to the Covid-19 pandemic, primarily supported projects that focused on formal responses to DVA (such as criminal justice agencies or established services). Therefore, the case example represented an exception to the main policy foci. However, the review did focus on interventions (and their effectiveness) and included substantial ‘impact’ orientated activities. The emphasis on staged milestones/ outcomes meant that the research adopted a rapid reviewing approach ‘in which systematic review processes are accelerated and methods are streamlined to complete the review more quickly’ (Tricco et al., 2017, p. 3). Steps were taken to ensure rigor of the process but the need to allocate resource to ‘impact’ activities may have detracted from the underpinning research. This can be considered ethically problematic if the funding is understood to inhibit the methodological rigour or compromise the integrity of the project (Suri, 2020). To address these concerns, DVA reviews can explicitly reflect on how research aims/ themes relate to wider policy/ social context and consider whether the emphasis on impact activities, central to field of DVA (Ellsberg & Heise, 2002), influenced the rigour of the review (and report in the limitations section).

#### Review aims

Pillar 2 of the *RIF* requires transparent reporting of research aims and encourages researchers to consider ‘who designed the aims and objectives’ (p.19). Systematic review practices and standards of conduct go further by stipulating that the perspectives and priorities of stakeholders need to ‘play an important role in defining the research question’ (Rees & Oliver, 2017; White et al., 2018b, p. 2). The case review developed, and prioritised research questions within the team and in collaboration with the wider Advisory Group members. Within the field of DVA research, there is recognition that the perspectives of victim-survivors need to be ‘placed front and centre in shaping the research agenda’ (Tarzia et al., 2017, p. 713) although it is difficult to judge the extent to which DVA systematic reviews involve stakeholders in setting the research question. A scoping review of health focused systematic reviews that reported stakeholder involvement did not identify any reviews from the field of DVA (Pollock et al., 2018). Indeed, whilst systematic review reporting guidelines require explicit reporting of the research aims (Page et al., 2021b; The Methods Coordinating Group of the Campbell Collaboration, 2016), there is no requirement for details to be provided about the process for generating research questions in the first place. This is ethically significant as it is essential that readers are able to judge whether review questions are appropriate (Oliver et al., 2017) and the investment of resources in a review is worthwhile (Suri, 2020).

#### Methods Reporting

Transparent reporting of methodology is an inherent, defining feature of the systematic review approach (Gough et al., 2017b). There are a number of standards that guide the reporting of systematic reviews to ‘help systematic reviewers transparently report why the review was done, what the authors did, and what they found’ (such as M. Campbell et al., 2020; Page et al., 2021b Abstract). The case review adhered to both conduct and reporting standards in the field of systematic reviews and so met, and exceeded, the expectations of transparency/ accountability set out in the *RIF*. Without a systematic overview of all reviews in the field of DVA, it is difficult to gauge the transparency of reporting in this field. Indications from specific reviews of reviews, such as interventions with young people (Kovalenko et al., 2022), suggest that improvements in reporting/ transparency would be welcome. Yet, journals that publish systematic reviews in DVA, such as *Journal of Family Violence*, strongly encourage authors to adhere to reporting standards. Therefore, where possible DVA reviews should aim to follow and adhere to established reporting standards.

#### Outputs

The *RIF* stipulates that research teams should adhere to ‘standard authorship guidelines’ and allocate ‘the roles and responsibilities of authors, including both academic and non-academic partners’ (Women’s Aid, 2020, p. 9). Authorship was offered to all researchers and members of the Advisory group, guided by the CrediT taxonomy (Brand et al., 2015) and in line with expectations associated with social science authorship (British Sociological Association, 2001). Explicit consideration of authorship, contribution and order is particularly important for systematic reviews, where there are typically large research teams. Indeed, publishers of systematic reviews, such as the Campbell and Cochrane Collaborations, require authors to specify their individual contributions to the research (Higgins et al., 2022). Yet, standards of review conduct and reporting do not always stipulate the rights or responsibilities of authors (such as White et al., 2018b). The case example discussed authorship of outputs with the Advisory group, proposing to recognise diverse contributions and provide scope for all members to be listed as article authors alongside the core research team. However, named authorship may not always be possible and/ or appropriate in the field of DVA. First, some publishers stipulate the requirements for authorship which may preclude the inclusion of diverse contributors/ Advisory group members (such as the Campbell Collaboration that use International Committee of Medical Journal Editors, n.d.). Second, named authorship may not be feasible and/ or appropriate for all non-academic members. Individuals with lived experience of DVA may be unable or reluctant to be named on written outputs. Third, whilst authorship may be diverse, the writing-up of research primarily remains the responsibility of the academic who may struggle to faithfully represent the perspectives of all contributors (Silverio et al., 2020). Therefore, the ethical issues surrounding authorship allocation in the context of DVA require sensitive consideration.

The transparency/ accountability identified in the *RIF* may be facilitated by open access publications. Open access refers to freely accessible research reports and has been shown to provide positive impacts in terms of disseminating research to non-academic groups and advancing citizen science projects (Tennant et al., 2016). Within the systematic review community, there have been definite moves towards open access publications (such as Campbell Systematic Reviews) and there is an ethical imperative to make publicly funded research available. Yet, without infrastructure to curate and support public access and use of reviews, current processes remain insufficient (Gough et al., 2019). Moreover, as the *RIF* highlights, open science practices also raise a number of ethical challenges in the field of DVA such as protecting privacy, safety and confidentiality of primary research participants (Campbell et al., 2019). Without assessing the ethical standards of included studies, systematic reviews may inadvertently contain studies with ethical insufficiencies (Vergnes et al., 2010) and so potentially amplify such research through open publishing.

### Pillar 3: Equality, Human Rights and Social Justice

The *RIF* ‘recognises the importance in the research process of being aware, and naming, issues linked to equality, human rights and social justice’ (Women’s Aid, 2020: 10). There is growing recognition that systematic reviews can help to explore or advance issues related to equality and social justice (such as Dukhanin et al., 2018; Perera et al., 2022). This resonates with the field of DVA, where research is highly valued for its potential to contribute to improving efforts to tackle abuse and improve outcomes for victim-survivors (Green & Morton, 2021; Spalding et al., 2015). Fitting within this tradition, ‘working towards improving outcomes for victim-survivors of DVA’ was identified as a ‘guiding principle’ for the case review and so in alignment with Pillar 3. Two further ethical issues surrounding equality and social justice within the review process will be considered further: researcher reflexivity and representing diverse perspectives.

#### Positionality and Reflexivity

The formation of the core research team included researchers of different ages, ethnicities, and experiences. The *RIF* implies that researcher demographics and experience influence the research project and should be considered in the design stages: ‘Researchers will bring different types of knowledge and experience to the research process’ (Women’s Aid, 2020: 10). Yet, the *RIF* does not specify a role for ongoing reflexive research practices. In the systematic review community, the importance of positionality or reflexivity in the review process is rarely acknowledged. Quality appraisal tools used in systematic reviews of qualitative research typically include assessment of positionality (Critical Appraisal Skills & Programme, 2018) but reflexivity statements are not part of reporting or conduct standards for systematic reviews (such as White et al., 2018a). Meta-narrative systematic reviews present an exception where one of the guiding principles includes reflexivity ‘throughout the review, reviewers must continually reflect, individually and as a team, on the emerging findings’ (Wong et al., 2013). Indeed, informed subjectivity and reflexivity are important for the systematic review process (Suri, 2008, 2020) and decisions about synthesis have been found to be influenced by the skills and perspectives that researchers bring to the review (Lorenc et al., 2016). Within the case review, one member of the team had no experience of research in DVA whilst others had lived experience of DVA. These backgrounds were openly discussed within the team but there was limited discussion about the role played by our experiences or knowledge in shaping the review. There are attempts to begin to examine the diversity of review teams and the role of authors’ demographics in influencing research questions and processes (Qureshi et al., 2020). Reflexivity is a necessary strategy to consider how familiarity/ experience influences the researcher (Berger, 2015) and is critical for collaborative DVA research (Linabary et al., 2021). Therefore, there is an ethical imperative to embed reflexivity in the conduct and reporting procedures of systematic reviews.

#### Representing Diverse Perspectives

The *RIF* highlights several intersecting factors that influence experiences of abuse and so recommends that research acknowledge diverse perspectives/ groups of victim-survivors (Women’s Aid, 2020: 18). This responds to wider concerns that DVA research is largely focused on particular groups, white poor women, at the expense of others, such as women of colour or male victims (Bent-Goodley, 2005). The case example was alert to these debates and took steps to ensure representation of different populations and/ or viewpoints. First, the composition of the Advisory group aimed to ensure representation of different perspectives based on ethnicity and geographical locations but potentially included other dimensions of difference too (such as social class). Demographic information for stakeholders was not explicitly collected but, just like primary research, may have some value for seeking to ‘understand whose perspective might not be represented and to understand the nuances of DVA across a variety of groups’ (Women’s Aid: 2020: 8). Whilst diverse stakeholders bring welcome contributions to review methods, it is also important to recognise heterogeneity within groups (Rees & Oliver, 2017) and the necessity of an intersectional approach (Green & Morton, 2021). This means that reviewers should explore different perspectives within, for example, racial and ethnic groupings (Ragavan et al., 2020) and whether/ how these shape priorities alongside other social characteristics.

Second, the review purposively sought studies that included marginalised voices. The search strategy included ‘grey literature’ sources, targeting organisations that were known to focus on DVA in diverse groups in the UK and globally (Schucan Bird et al., 2022) ). Searching for studies that represent a range of ‘contextual configurations and viewpoints’ is ethically defensible (Suri, 2020, p. 47) and did result in a broader sample of studies in the case review (e.g., identifying research with indigenous communities that was not found otherwise). However, with few curated libraries or databases of grey literature studies, significant resources and creativity are required to develop effective search strategies, drawing on the specialist knowledge of stakeholders (Mahood et al., 2014). In the case review, researchers from the wider DVA sector played a crucial role in identifying potential resources that house research (such as https://vawnet.org/) and organisations that may publish relevant applied/ non-governmental research.

Third, the data extraction tools collected data about the characteristics of the populations in the primary studies, including gender, ethnicity, age, migrated populations, and length of exposure to DVA. This allowed for the disaggregation of data on many characteristics specified in Pillar 3 but not all (i.e., disability and neurodiversity) (Women’s Aid, 2020: 9). However, sampling in the primary studies rarely included minoritized groups, such as first-generation migrants, and the authors rarely explored culture/ ethnicity in their analysis. Gaps in the primary data about the social identities of DVA victim-survivors inhibits the potential of researchers and systematic reviewers to analyse intersectionality and multiple perspectives (Cullen et al., 2021). Indeed, there are attempts to encourage studies to evaluate interventions against a number of equity measures (O’Neill et al., 2014). In DVA reviews that identify a small or limited evidence base, novel review methods (Huntley et al., 2020) or additional primary data collection (with participants who can represent missing voices) (Green & Morton, 2021) may serve to ensure comprehensive understanding. Developing such strategies in reviews is particularly important due to their role in informing policy and practice decisions (Suri, 2008).

### Pillar 4: Engagement

Pillar 4 of the *RIF* highlights the importance of collaborative research in DVA. This Pillar foregrounds the contributions of non-academic partners in the research process, with clearly defined roles and responsibilities (Women’s Aid, 2020: 12–13). Systematic reviews are naturally aligned with Pillar 4, as inherently collaborative pieces of research, with the potential for varying degrees of ‘user involvement’ in undertaking the review (Pollock et al., 2018; Rees & Oliver, 2017). This section will examine collaboration between academics and researchers from a domestic abuse organisation, and then consider stakeholder engagement.

#### Research Collaboration

The case review was inherently collaborative, designed and delivered by a team of university-based academics and specialist DVA researchers from a domestic abuse organisation. This collaboration was embedded in the design/budget of the project to enable meaningful contribution from all parties and build individual, team and organisational capacity for systematic reviews (Oliver et al., 2015). Collaborative systematic reviews in DVA raise several ethical issues. First, collaborative projects need to consider the relationships and distribution of power between different project contributors. Power distribution in the research process is a central concern for primary DVA research which seeks to minimize power differentials between the researcher and the researched (Bender, 2017). Whilst there are no research participants in systematic reviews, the distribution of power amongst the review team is arguably an important ethical issue. Standards of conduct for reviews have hitherto provided scarce guidance about collaborative working and power distribution across review teams (e.g., White et al., 2018) but shared ownership/ contribution to the review methods is part of the ethical decision-making process (Suri, 2020). In the case review, the academic team/ institution held the contract with the funder with one researcher from the domestic abuse organisation named as Co-investigator to recognise that ‘partners in research are equally able to shape the work’ (Women’s Aid, 2020: 13). The distribution of resources (time and funds), however, meant that the project was primarily led by academics. Yet, there were explicit attempts to integrate all expertise and share power in decision-making to generate good methodological decisions in the review (Oliver et al., 2017). Weekly meetings, for example, served to ensure that different perspectives were voiced and discussed.

Second, all collaborators may potentially bring (intentional or unconscious) allegiances or interests to the review process. Stakeholder groups may bring particularly strong vested interests to the topic (Rees & Oliver, 2017) whilst researchers often under-estimate their own vested interests (such as ideological or reputational) (Montgomery and Bell, 2021). The influence of these interests on the methods and outcomes of the review should be subject to ethical consideration (Suri, 2008). Collaborative reviews may potentially spotlight these different vested interests and/ or improve the management of them (Montgomery and Bell, 2021). The potential for conflicting interests in DVA research is not directly addressed in the *RIF*, or wider discussions of ethics (Bender, 2017; Ellsberg & Heise, 2002; World Health Organization, 2001), but crucial for collaborative projects. The case review included researchers with varied interests, potentially incompatible, which were managed through explicit discussion within the team and transparent reporting. Each member of the research team declared conflicts of interest in the published protocol (Schucan Bird et al., 2022) which adhered to the standards specified by systematic review guidelines (Higgins et al., 2022). However, such guidelines need to be expanded to encourage researchers to recognise all forms of vested interest and normalise reflexivity statements (Montgomery and Bell, 2021).

#### Stakeholder Engagement

Systematic reviews, like other pieces of research, are subjective and ‘need to draw on a range of perspectives to produce a rounded piece of work’ (Rees & Oliver, 2017, p. 27). Involving ‘stakeholders’ (those who may be affected by the work and/ or have a stake in the issue) in the systematic review process is a means of doing so. Stakeholder engagement in reviews of DVA raise various ethical and methodological issues. The decision to include stakeholders as participants in the review process can be understood as an ethical choice: embedding the voice of victim-survivors and relevant non-academic partners in the research is essential for ensuring relevance and maximising the benefits of DVA studies (Ellsberg & Heise, 2002; Women’s Aid, 2020). The methods used to engage stakeholders involves multiple ethical considerations and this section will only focus on the ethical selection and involvement of stakeholders in the review.

Systematic reviews involve a diverse range of potential stakeholders in the research process (Rees & Oliver, 2017) and victim-survivors, service providers and non-governmental organisations are highlighted as important collaborators specifically for DVA research (Women’s Aid, 2020). The case review identified a diverse group of potential stakeholders in order to represent different types of knowledge/ experience in the review process (Rees & Oliver, 2017). The selection of the membership was then based on professional networks. Whilst the use of personal contacts is a common method for recruiting stakeholders to reviews (Pollock et al., 2018), this may also be ethically problematic if it means that particular perspectives are unknown/ inadvertently overlooked and so excluded from the review (Green & Morton, 2021; Suri, 2008). A representation framework may serve as a helpful tool for mapping out potential groups from which to invite representatives (Smith et al., 2009). The case example did, however, actively seek stakeholders that could represent perspectives of minoritized groups and different geographical locations in recognition of the ‘intersecting structural inequalities’ that underpin experiences of abuse (Women’s Aid, 2020: 10, Pillar 3).

The domestic abuse organisation involved in the review acted as gatekeepers for the recruitment process, considered ‘good practice’ (Women’s Aid, 2020: 5. Pillar 1), to ensure that individuals with lived experience had access to support services throughout the research. Similarly, frontline services known to them were identified and approached. Six experts/ scholars in DVA were approached by the PI, who were known through professional contacts, of which two agreed. Despite enthusiasm for the project, two experts were unable to be involved due to lack of resources/ capacity and a further two did not respond to the request. Indeed, this underlines the importance of ensuring that organisations are ‘costed in funded bids to cover their time and involvement’ (Women’s Aid, 2020: 13). Funding was allocated to individuals with lived experience and frontline services, at a rate that exceeded the recommended rates for involvement in health research (National Institute for Health and Care Research, 2022), but not for the involvement of wider DA organisations or experts in the field. This raises several ethical issues. First, whilst scholars/ experts were invited to contribute to written outputs (i.e., academic article), there are further considerations concerning the capacity/ time investment of these groups and appropriate incentives and/ or rewards for participation. Such details need to be explicitly considered to ensure transparency (Pillar 2) although systematic reviews rarely report details of financial or other compensation for stakeholder involvement (Pollock et al., 2018). Second, a fuller discussion needs to consider the benefits of involvement for individuals with lived experience, aside from financial compensation (Fontes, 2004), and the potential risk of the commodification of stakeholder engagement (Carr, 2019). Third, ethical DVA research places victim-survivor perspectives at the heart of the project (Women’s Aid, 2020). By involving a wide range of stakeholders, not only victim-survivors, in DVA reviews (including the case example) there is a risk that the contributions of individuals with lived experience will be diluted. It is therefore important to consider the balance of different knowledges and perspectives in the review team (Oliver et al., 2017).

### Pillar 5: Research Ethics

The *RIF* promotes the value of independent ethical reviews of research plans/ protocols and the importance of Research Ethics Committees (RECs). The case review adhered to Pillar 5 by submitting an ethical application to the institution’s panel for reviewing research ethics. The institutional process, however, did not require the PI to specify or reflect on the ethical issues associated with the systematic review process. This is common practice as ethical review boards do not typically include guidelines for systematic reviews (Suri, 2020). However, this experience illustrates concerns raised by social scientists about the value and utility of institutional ethical review procedures (Hunter, 2018). Critics suggest that RECs primarily focus on enforcing compliance with ethical regulations rather than enabling reflection about ethical practices in research (Allen & Israel, 2018). Further, there are specific concerns that the complex ethical dilemmas faced by researchers in DVA research are rarely addressed in the ethical guidelines of RECs (Downes et al., 2014). These challenges, not acknowledged or addressed by the *RIF*, pertain to numerous issues including a lack of understanding of how DVA services operate and a resistance to working with vulnerable groups, to which victim-survivors often belong (Green & Morton, 2021). The *RIF* could therefore be enhanced by recognising such challenges and providing guidance/ recommendations for RECs. The lack of ethical guidance for systematic reviews (Suri, 2020) further compounds the challenges associated with DVA as RECs are not equipped with a framework for guiding ethical practice in this methodology.

To better consider the ethical implications of systematic reviews in DVA, RECs/ institutional frameworks could be adapted in several ways. First, a regulatory system could be tailored to social science with methodological and subject specialists, such as systematic reviewers or DVA experts, residing on RECs (Hunter, 2018). Second, translation of ethical academic research (such as this paper and Suri, 2008, 2020) into the practice of RECs could support local and national frameworks for assessing systematic reviews. Third, the ethical priorities identified by DVA researchers can be used to guide assessment of review methods in this field. The centrality of victim-survivor voice in DVA research (Ellsberg & Heise, 2002; Women’s Aid, 2020) suggests that RECs should assess the presence, role and contribution of victim-survivors in systematic reviews whether as researchers, stakeholders and/ or authors/ participants in primary research studies. This could constitute part of broader consideration of ethical issues associated with how voices/ perspectives of different groups are included in research synthesis (Suri, 2008). Further, the significance of ethics in DVA research means that REC assessment of systematic reviews should also consider the methods used by reviewers to appraise the ethical quality of included studies. Whilst assessing the ethics of included studies is not typically undertaken (Vergnes et al., 2010), such scrutiny could serve to develop and improve practice.

## Limitations

To our knowledge, this is the first attempt to identify ethical and methodological issues that sit at the intersection of DVA research and systematic reviewing. The findings reported above need to sit alongside the following limitations. First, the analysis does not comprehensively consider the ethical or methodological issues of systematic reviews, of which there are many (Suri, 2020), but sketches out a selection of these debates. The dynamic nature of ethical considerations mean that these debates are potentially subject to continuous reinterpretation (Burgess-Proctor, 2015) and the conversations need to be ongoing. Second, the findings and recommendations are drawn from a single review (with multiple components). The case example is therefore not representative of all types of review and so the different approaches (such as meta-narrative reviews or cost/ economic evaluation reviews, see Gough and Thomas, 2017; Munn et al., 2018) may generate new or alternative ethical and methodological issues. The claims made in this article therefore need to further explored in other types of systematic reviews in DVA. Third, whilst the *RIF* has served as a useful tool for highlighting ethical considerations in DVA research, it also warrants several enhancements. (1) to ensure transparency and accountability (Pillar 2), the methods used to develop the framework should be reported. There are examples of ethical frameworks from the field of gender and violence that do so (such as World Health Organisation, 2021). (2) there are minimal attempts within the Pillars to make explicit links to wider ethical debates within the field of DVA and beyond. There are, for example, vigorous debates about the role of Research Ethics Committees in higher education (Hunter, 2018) that should be acknowledged and considered in Pillar 5. (3) the *RIF* is based on the ethical premise of ‘do no harm’ (Women’s Aid, 2020, p. 5) without discussion of this underpinning philosophical tradition (and rationale for this choice) or any others. Engaging with and identifying moral and philosophical frameworks that underpin our research is arguably part of the ethical endeavour (Suri, 2020). (4) Whilst recognising that ‘victim-survivor perspectives should be present at the outset of the research endeavour’ (Women’s Aid, 2020, p. 3 and Pillar 4: Engagement), the *RIF* did not explicitly involve victim-survivors in the development of the guidelines. There are wider examples of co-creating research integrity guidelines (Labib et al., 2022) that serve to illustrate potential methods and demonstrate value of integrating diverse perspectives.

## Conclusion

This paper has examined ethical and methodological issues at the intersection of DVA scholarship and systematic reviews to establish a new agenda for research practice and governance. Table 1 identifies priorities for researchers to enhance the ethics of systematic reviews in the field of DVA, organised in line with the Pillars of the *RIF*. The transparent and rigorous methods associated with systematic reviews mean that the review process is arguably primed to integrate stronger ethical standards of conduct in other fields too. Systematic reviewers (individuals, teams, and the wider review community) are encouraged to actively engage with ethical issues at each stage of the review process, including consideration of reviewer positionality and reflexivity.

**Table 1:** Recommendations for systematic reviewers, based on the Pillars of the *Research Integrity Framework*

*RIF* Pillar	Research practices to enhance the ethics of systematic reviewing in DVA
1. Safety and well being	• Identify and address the safety and wellbeing needs of reviewers and all stakeholder groups • Ensure specialist training for DVA reviewers including introduction to DVA and strategies of self-care/ well being • Seek consent from stakeholder groups throughout the review process • Assess the ethics of included studies
2. Transparency/ Accountability	• Report funder/ commissioner of review and explicitly consider the influence of funder requirements (such as ‘impact activities’) and wider policy agendas on the aims and methods of the review • Ensure stakeholder involvement in developing review aims/ questions and report methods for doing so • Adhere to systematic review reporting standards • Explicit consideration of contribution to, and authorship of, outputs with research team and wider stakeholders • Identify and address the ethical challenges associated with Open Access publication of systematic reviews in DVA
3. Equality, Human Rights and Social Justice	• Develop diverse review teams and Advisory groups (in terms of demographics and lived experience) • Undertake reflexive research practices; report positionality and include reflexivity statement in review • Adapt review methods to search for and report diverse perspectives, including marginalised voices
4. Engagement	• Design and budget for collaborative reviews with diverse groups of stakeholders • Take steps to minimise power imbalances across collaborators • Declare, publish, and reflect on the conflicts of interest of the entire project team
5. Research Ethics	• Systematic review and DVA specialists to input to, or reside on, RECs

Several implications arise from this agenda for research practice and policy. First, the systematic review community should continue to engage and widen discussion about ethics and systematic reviews. Additional analysis is required to comprehensively examine the ethical issues associated with each stage of the review process. This article has only highlighted a few overarching issues and so more thorough consideration, alongside wider debate with stakeholders, is required. Second, this analysis has extended the discussion of ethics in the field of DVA. Scholarship and guidelines surrounding ethical research practices should continue to evolve for all types of research in DVA (not just systematic reviews or secondary analysis). Third, institutions and research infrastructure (such as RECs) need to review current policies and procedures to ensure and enable the ethical conduct of systematic reviews in DVA (such as providing tailored support services to DVA reviewers). Fourth, systematic review organisations and the wider infrastructure (such as Cochrane and Campbell collaboration) need to engage with ethical debates and consider the revision of conduct and reporting guidelines.
